# Therapeutic effects of class I HDAC inhibitor CI‐994 in Alzheimer’s disease

**DOI:** 10.1002/alz.084770

**Published:** 2025-01-09

**Authors:** Wenyan Lu, Keiji Kawatani, Suren Jeevaratnam, Guojun Bu, Takahisa Kanekiyo, Yonghe Li

**Affiliations:** ^1^ Mayo Clinic, Jacksonville, FL USA

## Abstract

**Background:**

Alzheimer’s disease (AD) is the most common cause of age‐related dementia, and the presence of amyloid‐β (Aβ) plaques and tau‐containing neurofibrillary tangles is associated with the neurodegeneration and cognitive impairment in this incurable disease. Growing evidence shows that epigenetic dysregulation through histone deacetylases (HDACs) plays a critical role in synaptic dysfunction and memory loss in AD, and HDACs have been highlighted as a novel class of anti‐Alzheimer targets. Moreover, restoring Wnt/β‐catenin signaling, which is greatly suppressed in AD brains, is a promising therapeutic strategy for AD. CI‐994 is an orally active class I HDAC inhibitor that has undergone several phase II/III clinical trials on cancer treatment. Importantly, CI‐994 can cross the blood‐brain barrier and is a cognitive enhancer.

**Method:**

We evaluated the therapeutic potential of CI‐994 in human patient‐specific iPSC‐derived neurons and cerebral organoids with *APOE* ε4/ε4 genotype or carrying *MAPT* p.P301L mutation.

**Result:**

We found that CI‐994 is not only a potent class I HDAC inhibitor but also a novel modulator of Wnt/β‐catenin signaling, and that activation of Wnt/β‐catenin signaling by CI‐994 is associated with elevated Wnt co‐receptor LRP6 protein level. Moreover, CI‐994 increased synaptic protein levels, enhanced spontaneous synaptic firing and network formation, and decreased tau phosphorylation in iPSC‐derived neurons. Finally, CI‐994 significantly increased histone acetylation, activated Wnt/β‐catenin signaling, and decreased Aβ production and tau phosphorylation in AD patient‐specific iPSC‐derived cerebral organoids.

**Conclusion:**

Our findings indicate that CI‐994 is a novel potential therapeutic candidate for AD.

